# A Simulation Study on the Pacing and Driving of the Biological Pacemaker

**DOI:** 10.1155/2020/4803172

**Published:** 2020-05-21

**Authors:** Yue Zhang, Lei Zhang, Yong Wang, Kuanquan Wang

**Affiliations:** ^1^College of Computer Science and Technology, Harbin Engineering University, Harbin 150001, China; ^2^School of Computer Science and Engineering, University of New South Wales, Sydney, NSW 2052, Australia; ^3^Department of Diagnostic Radiology and Nuclear Medicine, University of Maryland, Baltimore, MD 21201, USA; ^4^School of Computer Science and Technology, Harbin Institute of Technology, Harbin 150001, China

## Abstract

The research on the biological pacemaker has been very active in recent years. And turning nonautomatic ventricular cells into pacemaking cells is believed to hold the key to making a biological pacemaker. In the study, the inward-rectifier K^+^ current (*I*_K1_) is depressed to induce the automaticity of the ventricular myocyte, and then, the effects of the other membrane ion currents on the automaticity are analyzed. It is discovered that the L-type calcium current (*I*_CaL_) plays a major part in the rapid depolarization of the action potential (AP). A small enough *I*_CaL_ would lead to the failure of the automaticity of the ventricular myocyte. Meanwhile, the background sodium current (*I*_bNa_), the background calcium current (*I*_bCa_), and the Na^+^/Ca^2+^ exchanger current (*I*_NaCa_) contribute significantly to the slow depolarization, indicating that these currents are the main supplementary power of the pacing induced by depressing *I*_K1_, while in the 2D simulation, we find that the weak electrical coupling plays a more important role in the driving of a biological pacemaker.

## 1. Introduction

The sinoatrial node (SAN) is the genuine pacemaker in the heart, which generates the electrical pulses automatically and initializes the heartbeats [1, 2]. The pulses propagate rapidly to the whole heart through the conduction network, such as the interauricular bundle and Purkinje fibers [3–5].

However, the dysfunction of the SAN would lead to the abnormality of the heart rhythm, which is the main cause of the arrhythmias and heart failure [6–9]. At present, the main solution to treat these SAN diseases is to implant electronic pacemakers [10], of which the defects include the short battery lifespan, infections, and thrombosis [11–15]. As a consequence, the biological pacemaker attracts the attention of the researchers to overcome the disadvantages [16–18]. One of the popular strategies is to create a biological pacemaker in the ventricle.

Nevertheless, under normal conditions, there is no automaticity for the ventricular myocytes, which are kept in the resting state unless stimulated by the external stimulus, such as the electrical pulse from the SAN [19]. The main difference between the ventricular myocyte and the SAN cells is that *I*_K1_ is intensely expressed in the former, but it is absent in the latter [20]. Namely, *I*_K1_ may play an important role in inhibiting the pacing, and nonautomatic ventricular myocytes might be converted into pacing cells by reducing *I*_K1_.

To test the conjecture, *I*_K1_ was downregulated below 0.4 pA/pF in the biological experiment, and the ventricular myocytes were able to generate electrical pulses automatically, which resembled those of the genuine pacing cells [21]. The result was validated by a model study of Silva and Rudy [22] on the existing Luo-Rudy guinea pig ventricular single cell model. When *I*_K1_ was suppressed by 81%, the ventricular myocytes presented pacemaker activities automatically with a stable period of 594 ms. The research was also supported by another model investigation which was based on the TNNP human ventricular myocyte model developed by ten Tusscher and Panfilov. When *I*_K1_ was downregulated by 92%, the ventricular myocytes generated electrical pulses automatically without external stimulus [23]. After analyzing the modified PB human ventricular myocyte model, Kurata et al. found that the automatic electrical pulses of the myocyte emerged when *I*_K1_ was suppressed to 15.4% [24].

The consistent conclusions are also drawn in other biological experiments. By suppressing *I*_K1_ in the newborn Wistar rats' ventricle, Hu et al. observed the increasing beating frequency which was in accordance with their hypothesis that the ventricular myocytes could be converted into pacing cells by the inhibition of *I*_K1_ [25]. In the experiment of Kapoor et al., they found that when *I*_K1_ was substantially reduced, the ventricular myocytes were converted into the SAN cells and ectopic pacemaker activities were detected in the ventricle [26]. Even in the large-animal model of the complete heart block, after the reduction of *I*_K1_, the pacemaker activity also emerged in the ventricle; Hu et al. detected the pacemaker activities in the atrioventricular block porcine ventricle with the increasing beating rate after the suppression of *I*_K1_ [27].

The experiments and model studies suggest that *I*_K1_ plays a major role in the pacing of the ventricular cardiomyocytes. However, there are more than 12 channel currents in the ventricular myocyte. What role do the other currents play in the pacing that is induced by the reduction of *I*_K1_? In the study, based on the TNNP06 model, the pacing activity is inspired by inhibiting *I*_K1_ first, and then, the effects of other currents are investigated, respectively.

In addition, do the currents enhancing the pacing also ensure the driving of the biological pacemaker? In response to the aforementioned question, the effects of these currents and the weak electrical coupling on the driving are analyzed in the 2D model, respectively. Finally, the pseudo electrocardiogram (ECG) is computed to evaluate the functioning of the biological pacemaker.

## 2. Models and Methods

In this section, we introduce the models of the single cell and the 2D tissue and the method to compute the pseudo ECG, respectively.

### 2.1. The Model of the Single Cell

The well-established TNNP 2006 model of the human ventricular cells [28] is employed in the study, which is shown in

(1) $$\begin{array}{c} \frac{dV}{dt}=-\frac{I_{\text{ion}}+I_{\text{stim}}}{C_{\text{m}}}, \end{array}$$

where *I*_ion_ = *I*_Na_ + *I*_K1_ + *I*_to_ + *I*_Kr_ + *I*_Ks_ + *I*_CaL_ + *I*_NaK_ + *I*_NaCa_ + *I*_pK_ + *I*_pCa_ + *I*_bCa_ + *I*_bNa_.

In Equation (1), *V* is the transmembrane potential; *I*_ion_ is the sum of all the transmembrane ion currents; *I*_stim_ is the external stimulus current; *C*_m_ is membrane capacitance per unit surface area; and *I*_Ks_, *I*_Kr_, and *I*_to_ are the outward slow, rapid, and transient and rectifier potassium currents, respectively.

For the ventricular myocytes, *I*_stim_ in [28], Istim was set to -52 nS/pF to simulate the electrical stimulus from the SAN, because there were no pacemakers in the ventricular tissue, while in the study, *I*_stim_ is set to 0 in that there are electrical excitations from the biological pacemaker. And the aim of the study is to investigate whether the excitation from the pacemaker is strong enough to inspire the AP of the ventricular myocytes in the tissue.

In the model, all the currents are controlled by the maximal conductance. As a consequence, we modulate the currents by adjusting their corresponding maximal conductance in the simulation. In particular, because the nonautomatic ventricular myocytes could be converted into pacing cells by reducing *I*_K1_ [21–26], we derive the pacing cell model by depressing *G*_K1_, the maximal conductance of *I*_K1_.

### 2.2. The Model of the 2D Tissue

**Figure 1:**
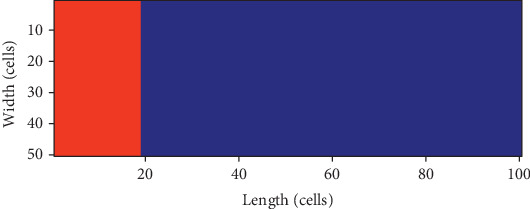
The 2D tissue: the red area—the pacemaker; the blue region—the ventricular tissue.

In the simulation, as shown in Figure 1, the 2D tissue is 100 cells in length and 50 cells in width. The 25 columns of cells on the left are the endomyocardial myocytes which would be converted into pacing cells, and the 40 columns on the right are the epimyocardial myocytes, and another 35 columns in the middle are the midmyocardial myocytes.

**Figure 2:**
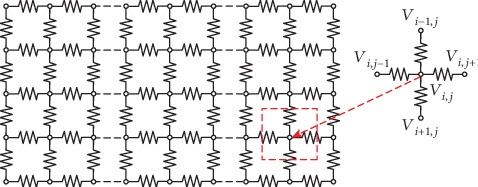
Electrical equivalent circuit of the 2D tissue in Figure 1.

The electrical equivalent circuit of the 2D tissue in Figure 1 is described in Figure 2.

In Figure 2, the small circles are the cells, and the resistance wires represent the electrical coupling (*D*) between cells.

The reaction-diffusion equation is adopted to describe the propagation of the electrical excitation wave in the 2D ventricular tissue, which is shown in Equation (2):

(2) $$\begin{array}{c} \frac{\partial V}{\partial t}=-\frac{I_{\text{ion}}+I_{\text{stim}}}{C_{\text{m}}}+D\Delta V, \end{array}$$

where *D* is the diffusion tensor with the normal value 0.154 cm^2^/s, which presents the electrical coupling in the tissue and describes the conductivity of the excitation; Δ is the Laplace operator; and the other parameters are the same as in Equation (1).

On the 2D level, Equation (2) could be discretized as the partial differential:

(3) $$\begin{array}{c} \frac{\partial V_{i,j}}{\partial t}=-\frac{I_{\text{ion}}+I_{\text{stim}}}{C_{\text{m}}}+D\left(V_{i+1,j}+V_{i-1,j}+V_{i,j+1}+V_{i,j-1}-4V_{i,j}\right), \end{array}$$

where *V*_*i*,*j*_ is corresponding to that in Figure 2. −((*I*_ion_ + *I*_stim_)/*C*_m_) is the reaction term, which is the same as that in Equation (1) and updated by the cell (*i*, *j*) itself. And *D*(*V*_*i*+1,*j*_ + *V*_*i*−1,*j*_ + *V*_*i*,*j*+1_ + *V*_*i*,*j*−1_ − 4*V*_*i*,*j*_) is the diffusion term, which describes the effect of the four surrounding cells on the cell (*i*, *j*). Both the reaction and diffusion terms contribute together to the change of the action potential of the cell (*i*, *j*).

### 2.3. Pseudo ECG Computing

To simulate ECG, a virtual electrode is placed at (*x*_0_, *y*_0_), which is about 1.5 cm away from the right end of the 2D tissue in Figure 1. The pseudo ECG is calculated as follows [14]:

(4) $$\begin{array}{c} \text{ECG}=\int \frac{\sigma \nabla V\cdot \overset{⃑}{r}}{r^{3}}dS, \end{array}$$

where *σ* is a constant; ∇ is the gradient operator; *V* is the transmembrane potential; $\overset{⃑}{r}=\left(x-x_{0},y-y_{0}\right)$ is the vector from the electrode to the point (*x*, *y*) in the tissue; *S* is the area of the virtual tissue; and *r* = ((*x* − *x*_0_)^2^ + (*y* − *y*_0_)^2^)^1/2^ is the distance from the point (*x*, *y*) to the electrode.

The pseudo ECG formulation could be discretized as follows:

(5) $$\begin{array}{c} \text{ECG}=-\sigma \int \int \frac{1}{r^{3}}\left(\left(x-x_{0}\right)\frac{dV_{\text{m}}}{dx}+\left(y-y_{0}\right)\frac{dV_{\text{m}}}{dy}\right)dxdy=-\sigma \sum \sum \frac{1}{{\left({\left(x-x_{0}\right)}^{2}+{\left(y-y_{0}\right)}^{2}\right)}^{3/2}}\left(\left(x-x_{0}\right)\frac{V_{\text{m}}\left(x+∆x,y\right)-V_{\text{m}}\left(x-∆x,y\right)}{2∆x}+\left(y-y_{0}\right)\frac{V_{\text{m}}\left(x,y+∆y\right)-V_{\text{m}}\left(x,y-∆y\right)}{2∆y}\right)\cdot ∆x∆y. \end{array}$$

In our simulation, the time step is set to 0.02 ms and the length step is 0.15 mm; all the simulation time is more than 20,000 ms in order to obtain a stable state.

## 3. Results and Discussion

In this section, firstly, the automaticity of the ventricular myocyte is induced by depressing *I*_K1_; and then, the effects of other currents on the pacing are analyzed to elucidate which currents play the major supplementary role in the initiation of the automatic pacing and which ones in the rapid depolarization. Moreover, we investigate how the automatic cells excite the ventricular myocytes and how the pulses propagate in the tissue. The effects of the channel currents and the weak coupling on the driving of the pacemaker are probed, respectively. Finally, the pseudo ECG is adopted to evaluate the effect.

### 3.1. Effects of the Currents on the Automaticity of Single Ventricular Cell

**Figure 3:**
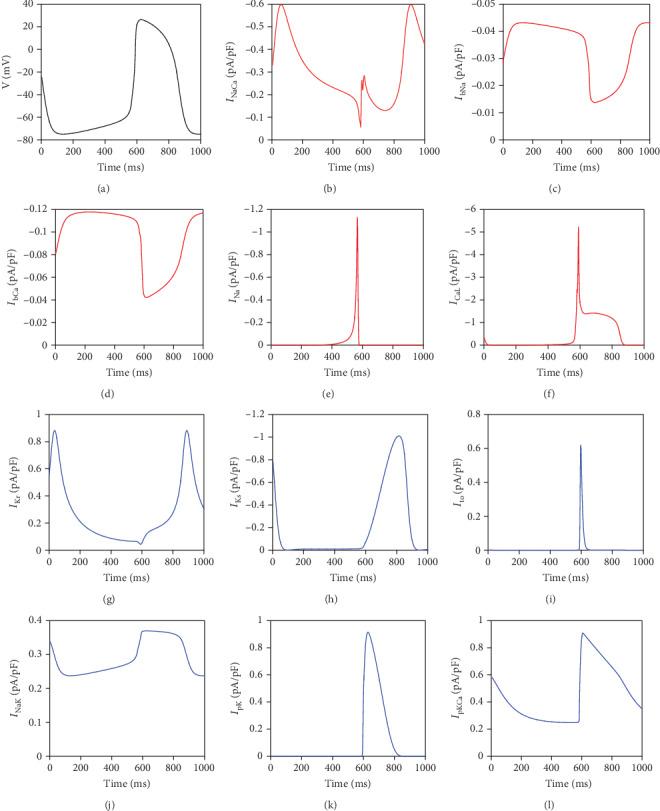
Simulated spontaneous AP in the TNNP ventricular cell model with *I*_K1_ reduction (*G*_K1_ was set to 0.05 nS/pF) and the underlying ion channel currents. (a) AP. (b) *I*_NaCa_. (c) *I*_bCa_. (d) *I*_bNa_. (e) *I*_Na_. (f) *I*_CaL_. (g) *I*_Kr_. (h) *I*_Ks_. (i) *I*_to_. (j) *I*_NaK_. (k) *I*_pK_. (l) *I*_pCa_.

As the previous work [23] suggests that the automaticity of the ventricular myocyte is generated when *G*_K1_ is less than 7.7% of the normal value, *G*_K1_ is reduced to 0.05 nS/pF (0.93% of the normal value) to convert the myocyte into the pacemaker cell, the AP of which is illustrated in Figure 3(a) as black curves. It is shown that the AP emerges automatically, with a slow depolarization phase followed by a fast depolarization phase which leads to a full AP.

And then, all the channel currents in Equation (1) are recorded and illustrated in Figures 3(b)–3(l). It is known that the negative currents contribute to the rise of the AP, while the positive currents depress the AP. In Figure 3, the positive currents are presented by the blue curves, and the negative currents by the red; what is more, because the positive or the negative only presents the direction of the currents, in order to describe the value intuitively, the vertical axis is reversed for the negative currents.

From Figure 3(a), it is easily seen that the AP increases slowly from 50 ms to 550 ms when the inward depolarizing currents of *I*_bCa_, *I*_bNa_, and *I*_NaCa_ counter-balance the outward repolarizing currents of *I*_Kr_, *I*_NaK_, and *I*_pCa_, leading to the generation of the automatic AP, while the rapid depolarization lasts from 550 ms to 600 ms, when the *I*_Na_ and *I*_CaL_ surge.

From Figure 3(b), it is revealed that the peak value of *I*_NaCa_ is at about 50 ms, when the slow depolarization begins; that is, *I*_NaCa_ may play a major role at the initial stage of the slow depolarization, while from Figures 3(c) and 3(d), the larger values of *I*_bCa_ and *I*_bNa_ continue from about 100 ms to 550 ms when the AP rises gradually, which infers that *I*_bCa_ and *I*_bNa_ might accelerate the slow depolarization.

From Figures 3(e) and 3(f), we could find that *I*_Na_ and *I*_CaL_ increase sharply from around 550 ms to 580 ms, which are corresponding to the rapid depolarization of the AP. This indicates that *I*_Na_ and *I*_CaL_ could be key factors of the rapid depolarization.

In summary, *I*_bCa_, *I*_bNa_, *I*_NaCa_, *I*_Na_, and *I*_CaL_ might play a positive role in the slow and rapid depolarization of the automatic AP.

In the following, to illustrate the effects of these currents more clearly, the currents will be discussed individually.

**Figure 4:**
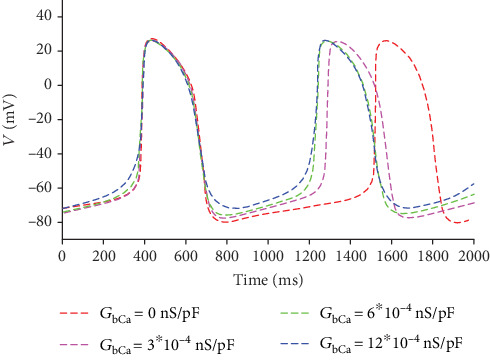
APs corresponding to different *G*_bCa_.

To investigate the role of *I*_bCa_ in the generation of automatic AP, we modulate the current by changing its channel conductance *G*_bCa_, which ranges from 0 nS/pF to 12∗10^−4^ nS/pF (the normal value is 5.92∗10^−4^ nS/pF). The APs corresponding to different *G*_bCa_ are shown in different colors in Figure 4.

From Figure 4, it is demonstrated that with the increase of *I*_bCa_, the period of the pacing decreases rapidly, and the minimal value of the APs improves. We may infer that the automaticity is enhanced by the growing *I*_bCa_ to an extent. The similar results could be gained by increasing *I*_bNa_ or *I*_NaCa_.

However, using the same method, we observe that the automaticity of ventricular cell decreases with the increase of the outward repolarization currents *I*_Kr_, *I*_NaK_, and *I*_pCa_.

**Figure 5:**
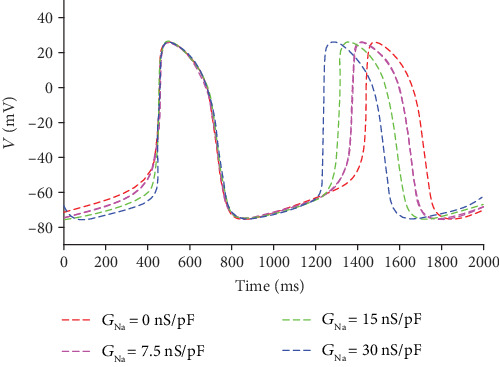
APs corresponding to different *G*_Na_.

*I*_Na_ is another current which might play an important role in the rapid depolarization. The current is modulated by its maximal channel conductance *G*_Na_ from 0 nS/pF to 30 nS/pF (the normal value is 14.838 nS/pF). The APs corresponding to different *G*_Na_ are shown in Figure 5.

From Figure 5, it is revealed that the pacing frequency increases with the growth of *I*_Na_. The period decreases from 986.3 ms when *G*_Na_ = 0 nS/pF to 783.3 ms when *G*_Na_ = 30 nS/pF. In the rapid depolarization stage (the 0 phase), the slope decreases with the reduction of *I*_Na_. The maximal velocity of the pacing depolarization is 12.59 V/s when *G*_Na_ = 30 nS/pF, while it is 4.95 V/s when *G*_Na_ = 0 nS/pF. In short, the increasing *I*_Na_ accelerates the pacing activity.

**Figure 6:**
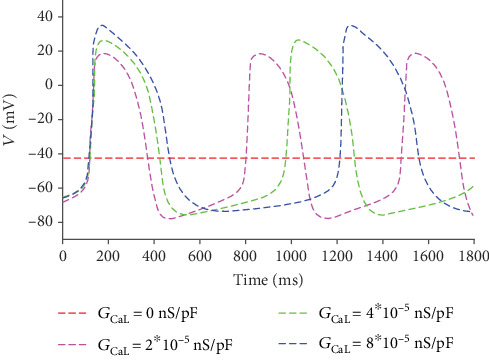
APs corresponding to different *G*_CaL_.

In the following, we investigate the effect of *I*_CaL_, another major active inward current during the rapid depolarization phase. In the simulation, the current is controlled by the maximal channel conductance *G*_CaL_, from 0 nS/pF to 8∗10^−5^nS/pF (the original value is 3.98∗10^−5^nS/pF). The APs corresponding to different *G*_CaL_ are described in Figure 6.

Figure 6 shows that *I*_CaL_ has a remarkable effect on the pacing. A smaller *I*_CaL_ leads to a lower peak potential. Moreover, the pacing activity fades completely when *I*_CaL_ is zero. The simulation results suggest that *I*_CaL_ plays a great role in ensuring the depolarization, which resembles the case in an early embryo heart, where *I*_CaL_ contributes mainly to the cardiac depolarization [29].

**Figure 7:**
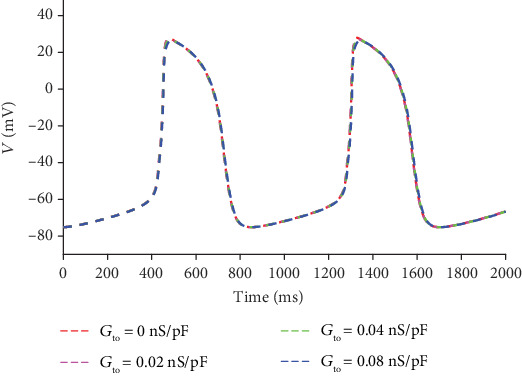
APs corresponding to different *I*_to_.

Similar investigations are conducted to investigate the role of *I*_to_ on the automatic APs. In the simulation, the maximal channel conductance *G*_to_ (the normal value is 0.073 nS/pF) is reduced from 0.08 nS/pF to 0 to modulate the current. The simulation results are shown in Figure 7, which demonstrates that the APs are almost the same corresponding to different *G*_to_. That is, *I*_to_ has a negligible effect on the automatic AP.

### 3.2. The Driving of the Pacemaker

In the section, the effects of the negative currents and the weak electrical coupling on the driving of the pacemaker are probed. The different kinds of biological pacemakers are created, respectively; and then, the generation and propagation of the automatic electrical pulses from the biological pacemakers are investigated to discuss the driving.

Firstly, *n* columns of the cells on the left of the tissue in Figure 1 are converted into pacing cells by setting *G*_K1_ = 0.05 nS/pF, and *n* is increased from 1 to 25 by a step of 2. Even though all the 25 columns of endomyocardial myocytes are converted into pacing cells, it is found that the pacemaker could not pace and let alone drive the ventricular tissue (Case 1).

According to the previous section, *I*_bCa_, *I*_bNa_, *I*_NaCa_, *I*_Na_, and *I*_CaL_ contribute to the automatic pacing of the single myocyte. As a consequence, *I*_bCa_, *I*_bNa_, *I*_Na_, and *I*_CaL_ in the pacemaker are increased by two times in order to enhance the driving of the automatic cells (Case 2). However, the pacemaker fails to pace and is at a similar stalemate as Case 1, even though all the endomyocardial myocytes are converted into pacing cells. That is, the driving capacity is not enhanced apparently by increasing the pacing currents.

**Figure 8:**
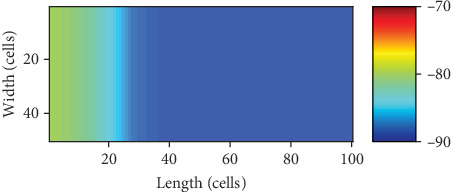
The stalemate between the pacemaker and the ventricular tissue.

In these cases, the potential of the pacemaker is depressed by the surrounding ventricular myocytes; meanwhile, the depressed potential of the pacemaker is not able to fire the surrounding myocytes to depolarize completely. As a result, the pacemaker and the ventricular tissue are in a stalemate as shown in Figure 8, of which the color bar ranges from -90 mV to -70 mV to show the detail more evidently. The maximum of the potential in the pacemaker is around -80 mV, which infers a failed depolarization.

Nevertheless, we discover that when *I*_bCa_, *I*_bNa_, *I*_Na_, and *I*_CaL_ in the whole tissue are increased by two times, the pacemaker, including 25 columns of pacing cells, could pace robustly and drive the whole ventricular tissue.

**Figure 9:**
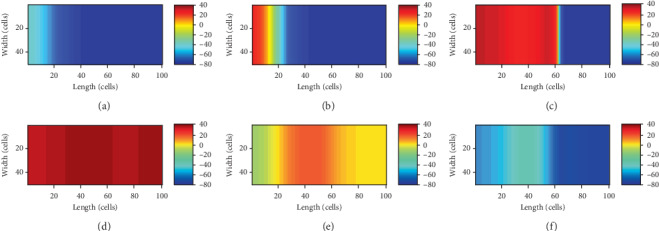
Snapshots of the pulse emerging in the pacemaker and propagating across the ventricular slice. (a) *t* = 190 ms; the pulse was emerging in the pacemaker; (b) *t* = 213 ms; the excitation was conducting to the border; (c) *t* = 223 ms; the wave was propagating in the ventricular tissue; (d) *t* = 300 ms; a slow repolarization state of the tissue; (e) *t* = 540 ms; a rapid repolarization state of the tissue; (f) *t* = 626 ms; the end of the repolarization.

Figure 9 shows the snapshots of the activation pattern across the tissue at varying timings.

In fact, it is arduous to increase *I*_bCa_, *I*_bNa_, *I*_Na_, and *I*_CaL_ by two times in the whole heart. The larger currents might result in corresponding heart diseases. However, comparing with Case 2 in which the pacemaker could not work, it indicates the positive effects of *I*_bCa_, *I*_bNa_, *I*_Na_, and *I*_CaL_ on the depolarization.

**Figure 10:**
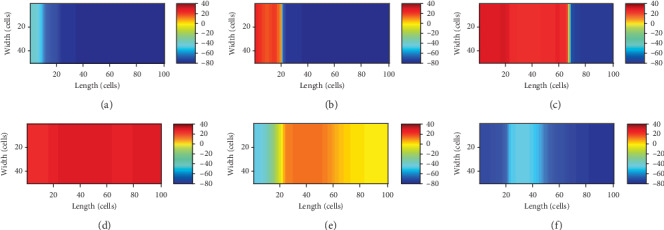
Snapshots of excitation wave propagating in the 2D tissue model. (a) *t* = 180 ms; the pulse was emerging in the pacemaker; (b) *t* = 200 ms; the excitation was conducting to the border; (c) *t* = 215 ms; the wave was propagating in the ventricular tissue; (d) *t* = 300 ms; a slow repolarization state of the tissue; (e) *t* = 480 ms; a rapid repolarization state of the tissue; (f) *t* = 550 ms; the end of the repolarization.

On the other hand, in the real heart, because of the weak coupling, there are only around 10,000 pacing cells to drive the whole heart consisting of about 10^10^ cells [30]. As a consequence, another method is utilized to create the biological pacemaker. The pacing cells are also obtained from the ventricular myocytes by depressing *G*_K1_ to 0.05 nS/pF, and the other currents are maintained as the normal values. The diffusion tensor *D* (the coupling) in the pacemaker is reduced to 0.09 of the normal, and only 23 columns of the endomyocardial myocytes on the left of the tissue are set to be the pacing cells. Then, it could be observed that the pacemaker paces robustly and the automatic pulses drive the whole ventricular tissue to generate excitation. The snapshots of the process are illustrated in Figure 10.

From Figures 9(b) and 10(b), 9(e) and 10(e), and 9(f) and 10(f), the significant difference of the two pacemakers is that the electrical boundary between the pacemaker and ventricular tissue is more obvious in the latter, where the pacemaker is protected better.

**Figure 11:**
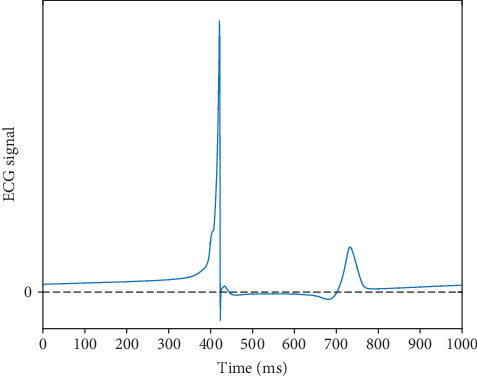
Simulated time course of the pseudo ECG in response to the conduction of excitation wave in the ventricular tissue.

In order to evaluate the global function of the pacemaker macroscopically, the pseudo ECG is computed as the excitation is propagating from the pacemaker to the tissue. And a whole period of ECG is presented in Figure 11.

The simulated ECG shows the typical features of the normal ECG with positive QRS and T waves; however, it remains positive after the T wave. This may be attributable to the more positive diastolic potential of the automatic cells (around -80 mV) as compared with the resting potential of the normal ventricular myocytes (about -86 mV). On the other hand, because of the weak coupling, the number of the pacing cells is reduced, attenuating the accumulation of the potential difference, making the signal after T wave closer to 0. In summary, the pacemaker created by weakening electrical coupling is more feasible and effective.

## 4. Conclusions

In the paper, the effects of the ion channel currents on the pacing and driving of the biological pacemaker are analyzed.

For pacing, our simulation data suggest that *I*_bCa_, *I*_bNa_, and *I*_NaCa_ play a major role in the initiation of the automatic depolarization, and *I*_CaL_ rather than *I*_Na_ makes the most contribution to the rapid depolarization of the APs. In contrast, the ion channel currents *I*_Kr_, *I*_NaK_, and *I*_pCa_, counter-balancing other depolarization currents, negatively affect the automaticity of the pacing cells. And *I*_to_ does not show notable effects on the automaticity.

While for driving, the weak electrical coupling plays a more important part. Withnormal coupling, a large number of pacing cells are required even though the negative currents *I*_bCa_, *I*_bNa_, *I*_Na_, and *I*_CaL_ are increased.

The results also reveal that it requires a critical size of an automatic cell region to drive the surrounding ventricular tissues. The pacing would be depressed if the pacemaker is small though the single cells present strong automaticity.

## Data Availability

The data supporting this research are from previously reported studies, which have been cited.
